# Perceived School Experience of Children and Adolescents With Type 1 Diabetes in the Kingdom of Saudi Arabia

**DOI:** 10.7759/cureus.44335

**Published:** 2023-08-29

**Authors:** Haila Alshelowi, Bilal Ahmad, Omer Bin Abdul Aziz, Hassan Badawi, Anjum Muhammad

**Affiliations:** 1 Department of Pediatrics, Qassim Armed Forces Hospital, Al-Qassim, SAU; 2 Department of Endocrinology and Diabetes, Qassim Armed Forces Hospital, Al-Qassim, SAU; 3 Department of Surgery, Qassim Armed Forces Hospital, Al-Qassim, SAU; 4 Department of Internal Medicine, Qassim Armed Forces Hospital, Al-Qassim, SAU; 5 Department of Dermatology, Pak Emirates Military Hospital, Rawalpindi, PAK

**Keywords:** mixed perception, perceived experience, diabetes school management, diabetes in adolescents, diabetes in children, insulin injection, school health services, type 1 diabtes, patients experience, patients’ perception

## Abstract

Background and objective: Type 1 diabetes mellitus (T1DM) is one of the most common chronic diseases in children globally affecting more than 1.2 million children worldwide. It is challenging to manage in children and adolescents, as it can have much more serious psychosocial impacts in these groups. The objective of this study was to investigate the perceived experience of children and adolescents with T1DM regarding the management of their condition while in school.

Methods: We used a cross-sectional study design with descriptive statistics and non-probability consecutive sampling in this work. This study was conducted at the Department of Pediatrics, Qassim Armed Forces Hospital, Al-Qassim, Saudi Arabia, from July 2018 to December 2018. In this study, we included 84 school-aged children and adolescents from various schools in the Qassim region of the Kingdom of Saudi Arabia who had T1DM and met the inclusion criteria. After we obtained written informed consent from the participants, they filled out a survey questionnaire about their perceived school experience while being a T1DM patient.

Results: Although most of the children believed that they were not prevented from managing their diabetes at school, most also believed that school personnel did not have adequate knowledge about diabetes.

Conclusion: In this study, adolescents and children with T1DM had mixed perceptions of their experience at school.

## Introduction

Type 1 diabetes mellitus (T1DM) is an autoimmune disorder resulting from the immune-mediated destruction of insulin-producing pancreatic beta cells leading to an absolute deficiency of insulin [1]. T1DM is one of the most common chronic diseases in children globally [2]. Studies have shown that T1DM incidence is increasing by 3-4% every year and presenting at a much younger age in children [3]. The Kingdom of Saudi Arabia (KSA), which is the largest country in the Arabian Peninsula, ranks seventh in global T1DM prevalence and fifth in incidence. The Kingdom has a population of 33.5 million, of which 26% is below 14 years of age [4]. Data from various studies reveal that, in recent years, there has been a significant surge in the prevalence and incidence rates of T1DM in the KSA, mainly among children and adolescents [5,6].

T1DM is challenging to treat in children and adolescents because of delayed diagnosis, increasing prevalence, early and late complications, and lack of resources for the management of this condition in school settings. Psychosocial issues and other factors, such as social behaviors, lack of knowledge, and understanding of the disease may interfere with glycemic control [7]. Recent research has shown that with the increasing prevalence of T1DM, addressing the relevant risk factors should be prioritized. For example, health programs and seminars can be used to educate mothers and parents of children and teenagers who are at risk of having diabetes mellitus [8,9].

In our study, we investigated the perceived experience of the children with T1DM regarding the management of their disease while in school. We focused on both the self-perception of the patients and the adequacy of school care regarding T1DM management.

## Materials and methods

We conducted this study at Qassim Armed Forces Hospital, Al-Qassim, Saudi Arabia, between July 2018 and December 2018 after obtaining approval from the Hospital Research and Ethical Committee (letter number #0985/QAFH/18). We used the non-probability consecutive sampling method to collect data on basic information such as demographics, educational level (e.g., primary, secondary, or intermediate), and their perception of their experience at school. We included female and male school-going children and adolescents aged five to 16 years in the study. All patients were currently using insulin to control diabetes. We excluded patients with other comorbidities (e.g., cardiac diseases or glycogen storage diseases) from the study. We obtained informed written consent from all patients who met the inclusion criteria before including them in the study.

We included in this study a total of 84 students with T1DM from different community schools. We gave the students a survey questionnaire (physical copy attached as Appendix) to fill out with questions regarding their perception of their experience at school as a diabetic patient. We explained each question to the study population before they began answering the questions.

Data analysis

We entered the study participants’ demographic data (e.g., age, gender, and school identification number) into a pre-designed proforma questionnaire (Appendix). Each question had four response options as follows: 1 = yes, 2 = sometimes, 3 = rarely, and 4 = no. We compared the data of different variables using SPSS version 20.0 (Armonk, NY: IBM Corp.). We used descriptive statistics to calculate the means and standard deviations (SDs) of quantitative data (e.g., age). We analyzed qualitative data (e.g., gender and frequency of replies to questions) using the chi-squared test.

## Results

**Figure 1:**
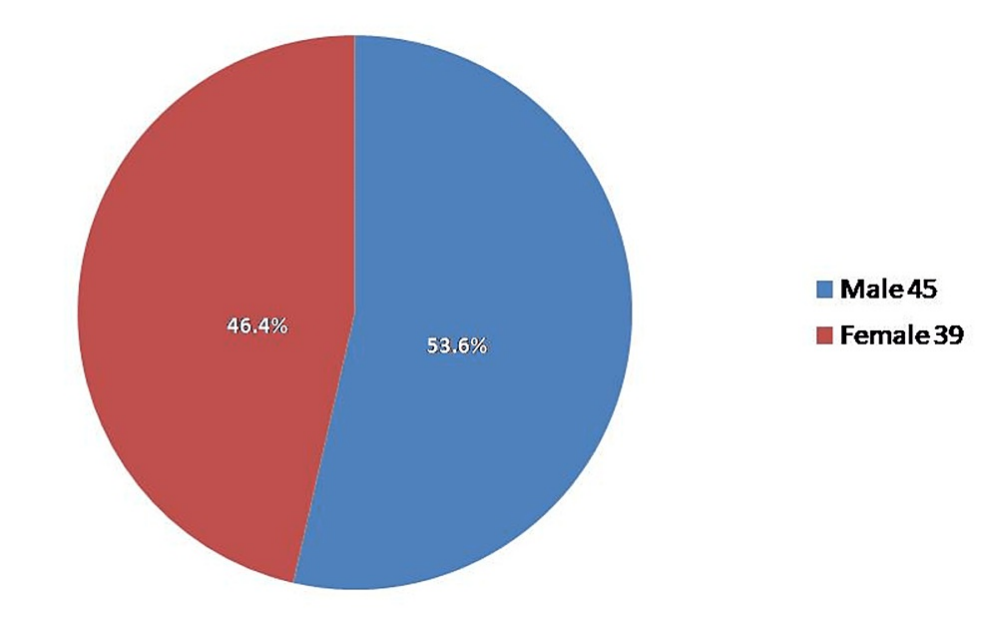
Gender distribution of the study population.

The population of the study comprised 84 patients who fulfilled the inclusion criteria. There were no droppedouts or lost subjects at any point in the study. Out of 84, 45 (53.6%) of the study population was male and the rest 39 (46.4%) were females (Figure 1).

A total of 42.9% (n=36) of the patients were from primary school, 27.4 (n=23) were from intermediate school and 29.7% (n=25) patients were from secondary school. The mean age of the study population was 10.7619±3.452 years. The mean age of male patients was 10.755±3.581 and female patients was 10.769±3.343 years. The mean age in the primary group was 7.33±1.45 years, intermediate group 12.08±0.79 years, and in the secondary group 14.48±1.96 years. Seventeen (20.2%) patients believed that they were treated differently in school as compared to other students, whereas 32 (38.1%) believed that they were not treated differently because of their diabetes (Table 1).

**Table 1:** Differential treatment at school due to diabetes.

Age group	Gender	Yes	Sometimes	Rarely	No	Total
Primary	Male	7	6	4	3	20
	Female	2	7	1	6	16
Intermediate	Male	2	2	1	6	11
	Female	2	3	2	5	12
Secondary	Male	0	4	1	9	14
	Female	4	4	0	3	11
Total		17	26	9	32	84

Only three (3.6%) participants had the perception that they were accused of using diabetes as an excuse for things such as missing their classes, sick leaves, and poor school performance. Out of these three participants, two were male and one was female. Sixty-one (72.6%) patients didn’t think that they were ever accused of using their disease as an excuse at school (Table 2). More than 2/3rd (n=58, 69%) of the patients claimed that they were not prevented from receiving treatment for their diabetes at school. The remaining 26 patients (31%) were of the opinion that they were either stopped sometimes or not allowed to receive their treatment at all at school.

**Table 2:** Accused of using diabetes as an excuse.

Age group	Gender	Yes	Sometimes	Rarely	No	Total
Primary	Male	1	2	2	15	20
	Female	1	0	3	12	16
Intermediate	Male	1	1	1	8	11
	Female	0	2	2	8	12
Secondary	Male	0	2	1	11	14
	Female	0	3	1	7	11
Total		3	10	10	61	84

**Figure 2:**
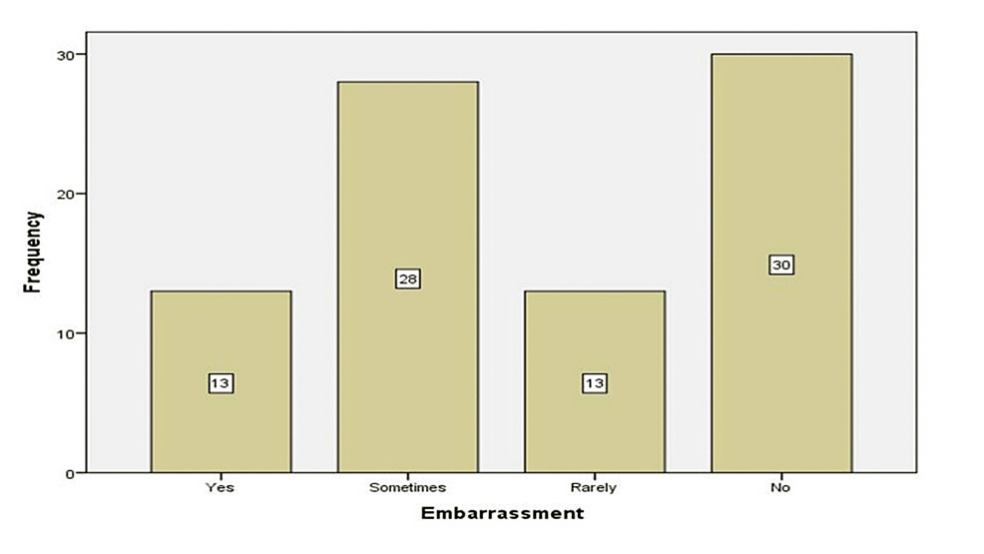
Participants who were embarrassed in front of their classmates due to diabetes.

A total of 64.3% of the participants felt embarrassed in front of their classmates at some point, whereas 35.7% of the participants did not feel embarrassed at all. A slightly higher proportion of female participants felt embarrassed as compared to male participants (66.7% vs. 61.5%), but this difference was not statistically significant (p>0.05). These students felt embarrassed not only because of their diagnosis of T1DM but also because of use of injections and their dietary requirements (Figure 2).

**Figure 3:**
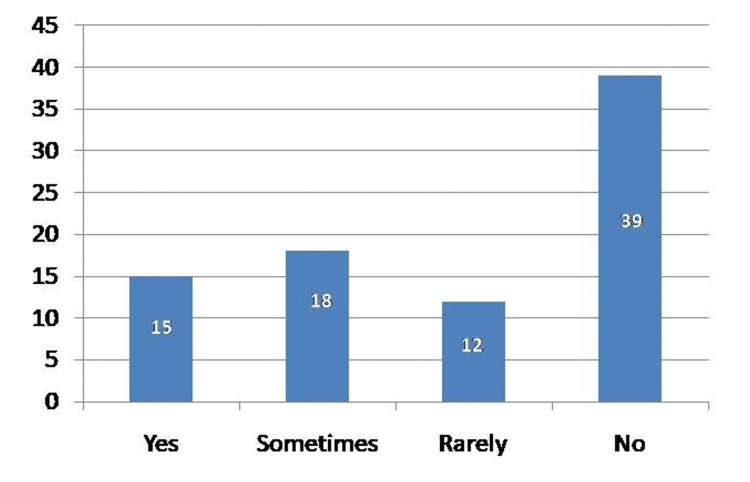
Perception of the school persons' knowledge about diabetes.

A higher proportion of female participants (n=5, 12.8%) felt a desire to quit school compared to male participants (n=3, 6.7%), but this difference was not statistically significant. A total of 70.2% of the participants had no desire to quit school (Table 3). Regarding school personnel knowledge, 46% participants felt that their school personnel did not know enough about diabetes with only 18% of the participants believing that the school staff knew enough (Figure 3).

**Table 3:** Children having the desire to quit school.

Age group	Gender	Yes	Sometimes	Rarely	No	Total
Primary	Male	3	2	2	13	20
	Female	2	3	1	10	16
Intermediate	Male	0	1	0	10	11
	Female	1	1	0	10	12
Secondary	Male	0	2	1	11	14
	Female	2	2	0	7	11
Total		8	11	4	61	84

**Figure 4:**
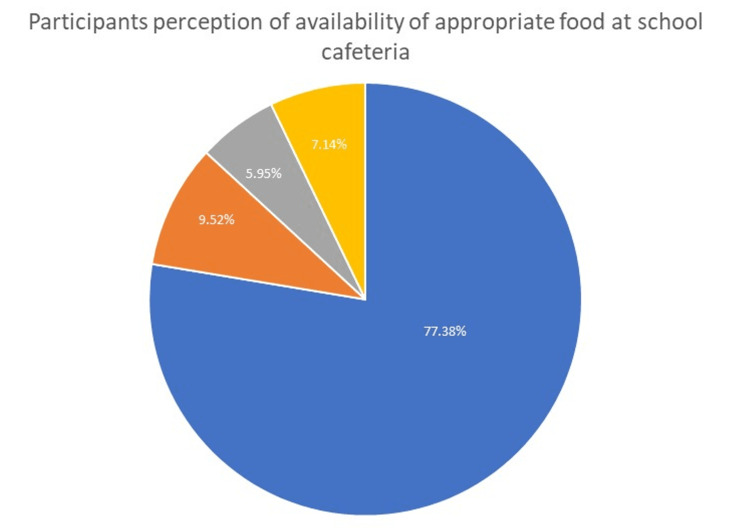
Proper food at school cafe.

Although 70% of the participants claimed that they were not prevented from managing diabetes at school, approximately 30% did feel that going to school prevented them from taking their medicine in some way. Due to the unavailability of appropriate food at school, 63.10% of the participants had to bring meals from home. A total of 77.38% of the participants thought that food appropriate for diabetic children and adolescents (e.g., sugar-free or low-carb items) was not served in the school cafeteria (Figure 4).

The study population had a mixed response when they were asked whether they had any help on returning from hospitalization due to illness: 29% claimed that they did get help from school personnel, whereas 39% claimed that they did not. A total of 77% of the participants did not have a proper diabetes care plan available in school. Although most participants felt embarrassed by low blood sugar or hypoglycemia (56%) and by taking medication (i.e., insulin injection at school) (48.3%).

## Discussion

T1DM treatment is very demanding and multipronged. Children with T1DM must manage a complex and challenging daily treatment regime, which can have many negative impacts on their lives [10]. Some Saudi Arabian studies found a higher prevalence of psychological problems and a remarkably increased risk of depression among female T1DM patients [11,12]. However, in the KSA, there is often less emphasis on addressing the psychosocial aspect of disease management among children and adolescents [12].

This study intended to examine the perception of children and adolescents with T1DM about their school experience. Although this may not be the first survey in the KSA to investigate this subject, its importance cannot be underestimated, as T1DM is considered one of the most common chronically occurring diseases in Saudi Arabian children and adolescents. The KSA currently ranks seventh in diabetes prevalence worldwide [1,13].

We focused only on the study participants’ perception of their experience at school. The study population comprised 53.6% male and 46.4% female participants, which, in accordance with studies by Blohme et al. and Cherian et al., showed a slightly higher prevalence in males [14,15]. Although this higher prevalence in males is typical, other studies conducted in the KSA showed a slightly higher incidence in females [5,16,17].

Only three (3.6%) participants were of the perception that they were accused of using diabetes as an excuse at school, of which two were male and one was female. Sixty-one (72.6%) participants did not think that they were ever accused of using their disease as an excuse at school. In the same way, 29% of children reported disparagement from their peers regarding diabetes, which is believed to affect their glycemic control [9].

Almost half of the patients (46.2%) felt that school personnel did not know enough about diabetes, with only 18% thinking that the school personnel knew enough. This lack of knowledge has been a big challenge observed in other studies as well. A school environment that is not properly prepared to manage students with diabetes puts these students at a high risk of complications [18]. In a study by Alaqeel, the results suggested that most teachers (247; 57%) had a moderate level of knowledge about diabetes [19], whereas 31.4% (136) and 11.5% (50) had good or poor levels of knowledge, respectively, similar to results reported by Amillategui et al. [20] and Moran et al. [21]. Participation from school personnel is a very important aspect of T1DM management in students, as the latter spend much of their time in school, and the former is expected to be knowledgeable and informed [22].

Fortunately, almost 70% of the participants claimed that they were not prevented from managing diabetes at school, but approximately 30% did feel that going to school prevented them from taking their medicine in some way, which is similar to the results reported by Jacquez et al. [22]. Insulin therapy should always be available at schools, as diabetic students must consume meals and snacks there. Moreover, managing sick-day diabetes when more insulin is needed due to sickness also necessitates the availability of insulin at schools, as emphasized in a 2019 study that found that most of the children with T1DM check their blood glucose levels at school, with some requiring insulin administration [23]. In our study, 70.2% of the participants did not want to quit school. However, only 35.5% of the participants never felt embarrassed in front of classmates because of their disease or treatment. Due to the unavailability of appropriate food at the school, 63.1% of participants had to bring meals from home. A total of 77.38% of the participants thought that proper food was not available in the school cafeteria, as seen in the studies by Moran et al. [21] and Jacquez et al. [22].

The study population had a mixed response when they were asked whether they received any help from school personnel on returning from hospitalization due to illness. Twenty-nine percent claimed that they did receive help, whereas 39% claimed that they did not receive any help, with similar results shown in other local studies [9,15,21,22]. Seventy-seven percent of the students did not have a proper diabetes care plan available in the hospital. Jacquez et al. claimed that 79% of primary, secondary, and intermediate schools did not have a diabetic care plan, which is recommended for diabetes management in schools as a tool to maintain good glycemic control and prevent acute complications, such as hypoglycemia and hyperglycemia [22,23]. Most of the participants felt embarrassed by low blood sugar (56%) and by taking medication at school (48.3%) at some point in time. However, despite these feelings of embarrassment, 57.1% of the participants did not feel disconnected from school.

Overall, despite all the shortcomings and problems, the study population had a mixed perception of their experience at school - 54.9% had a negative overall perception, only 25.38% had an absolute negative perception, and 45.1% did not have a negative perception of their experience at school, as evidenced by the fact that they did not want to quit school or feel disconnected (57.1%). The results of this study are contrary to those of some studies where patients or parents had a negative perception regarding the school experiences [7,22,23].

This study had several limitations. It mainly relied on patients’ reply/survey questionnaires about their perceived school experience rather than interviews or observational techniques. Another limitation was the small sample size, which could be increased in follow-up studies by including more schools and regions of the KSA. The cultural and individual biases could not be eliminated fully which is another limitation of this study.

## Conclusions

We found that the T1DM children and adolescents in our study had a mixed perception of their experience at school. The availability of qualified and trained school personnel for the management of children with T1DM was very limited, as most schools were perceived to not have a proper care plan. We noted that proper food was not available at the school cafeteria for such patients. Stakeholders could improve these children's circumstances with provisions of suitable food diets at their schools. More educational campaigns for students and teachers along with further research are needed, especially in the post-coronavirus disease (COVID) era.

## Appendices

Perception of children and adolescents with type 1 diabetes regarding their school experiences

Demographics of Patients

**Table 4:** Participants' demographic data.

Name/ID	Age	Gender	School (please √)
			Primary
			Secondary
			Intermediate

Questionnaire

**Table 5:** Participants' survey questionnaire.

1. Self-perception of treatment at school	Yes	Sometimes	Rarely	No
Are you treated differently at school?				
Are you accused of diabetes as an excuse?				
Have you been prevented from managing your diabetes?				
Have you been embarrassed in front of classmates?				
Did you ever feel like quitting school or not getting involved?				
2. Adequacy of school care for diabetes?				
Do you feel school personnel know enough about diabetes?				
Does the cafeteria have proper food?				
Do you have to take meal to school?				
Do you get help on returning from hospital/illness?				
3. Personal impact of diabetes				
Are you embarrassed by low blood sugar?				
Are you embarrassed by taking your medication?				
Do you ever have feelings of disconnectedness?				
